# Enhancing Reconfigurable Intelligent Surface-Enabled Cognitive Radio Networks for Sixth Generation and Beyond: Performance Analysis and Parameter Optimization

**DOI:** 10.3390/s24154869

**Published:** 2024-07-26

**Authors:** Huu Q. Tran, Byung Moo Lee

**Affiliations:** 1 Faculty of Electronics Technology (FET), Industrial University of Ho Chi Minh City, Ho Chi Minh City 700000, Vietnam; tranquyhuu@iuh.edu.vn; 2 Department of Intelligent Mechatronics Engineering and Convergence Engineering for Intelligent Drone, Sejong University, Seoul 05006, Republic of Korea

**Keywords:** cognitive radio (CR), ergodic rate (ER), gamma distribution, outage probability (OP), reconfigurable intelligent surface (RIS)

## Abstract

In this paper, we propose a novel system integrating reconfigurable intelligent surfaces (RISs) with cognitive radio (CR) technology, presenting a forward-looking solution aligned with the evolving standards of 6G and beyond networks. The proposed RIS-assisted CR networks operate with a base station (BS) transmitting signals to two users, the primary user (PU) and secondary user (SU), through direct and reflected signal paths, respectively. Our mathematical analysis focuses on deriving expressions for SU in the RIS-assisted CR system, validated through Monte Carlo simulations. The investigation covers diverse aspects, including the impact of the signal-to-noise ratio (SNR), power allocations, the number of reflected surfaces, and blocklength variations. The results provide nuanced insights into RIS-assisted CR system performance, highlighting its sensitivity to factors like the number of reflectors, fading severity, and correlation coefficient. Careful parameter selection, such as optimizing the configuration of reflectors, is shown to prevent a complete outage, showcasing the system’s robustness. Additionally, the work suggests that the optimization of reflectors configuration can significantly enhance overall system performance, and RIS-assisted CR systems outperform reference schemes. This work contributes a thorough analysis of the proposed system, offering valuable insights for efficient performance evaluation and parameter optimization in RIS-assisted CR networks.

## 1. Introduction

Cognitive radio (CR) technology has emerged as a solution to enhance spectral efficiency [1]. CR stands as a software-defined radio, offering a notable solution to address spectrum scarcity while simultaneously reducing power consumption for communication requirements [2]. In [3], the authors introduced dynamic spectrum access as a new paradigm within CR, highlighting dynamic spectrum access and CR as promising candidates to fully enable wireless technology implementation in industrial wireless communications for industrial systems and applications, as well as to address the limitations of spectrum scarcity.

Reconfigurable intelligent surface (RIS) technology is characterized as a method aimed at achieving spectrum- and energy-efficient transmission [4,5,6]. It comprises numerous passive reflective elements, each capable of altering the phase of the reflected wireless signal. This phase manipulation enables the controlled and advantageous modification of the wireless propagation medium without requiring external power or complex signal processing. Consequently, the utilization of RIS in advanced communication realms like 5G and 6G is extensively acknowledged [5,7]. Passive reflectors, such as RISs, operate by collecting wireless signals emitted by a transmitter and then redirecting them towards a receiver. This procedure bears the potential to augment both the strength and quality of signals. A RIS comprises a flat surface adorned with multiple tiny reflecting elements, referred to as metasurfaces or *M* elements (with *N* metasurface *M* elements). These elements can be flexibly adjusted to manage the characteristics of electromagnetic waves as they traverse through. Metasurfaces usually feature subwavelength resonant structures capable of modifying the phase, amplitude, and polarization of incoming waves. Through the precise manipulation of the reflection properties of each *M* element, an RIS can direct waves towards a specific direction, amplify or weaken them, or even generate entirely new beams [8]. The authors in [9] conducted an examination of the effectiveness of a multihop full-duplex (FD) relaying system assisted by RIS. In this setup, an intermediate FD relay is utilized to overcome the inherent far-field path-loss effect present in RIS communication links.

### 1.1. Related Works

To boost both spectral efficiency (SE) and energy efficiency (EE), the authors in [10] introduced multiple intelligent reflecting surfaces (IRSs) into a downlink multiple-input single-output (MISO) cognitive radio system (CRS). This setup involves a single secondary user coexisting with a primary network (PN) that includes multiple primary user receivers. In [11], the authors proposed using an intelligent reflecting surface (IRS) to aid data transmission for secondary users within a multiple-input multiple-output (MIMO) CRS. The authors in [12] delved into the augmentation of spectral efficiency by combining RISs with CR technology. In [13], the authors devised an optimization framework to facilitate the symbiotic operation of a multiuser CR network (CRN). The study in [14] explored the utilization of RISs to enhance both the physical layer security and data transmission in underlay CRNs. This CRN comprises a PN based on non-orthogonal multiple access (NOMA) and a secondary network (SN) based on RIS, both sharing the same spectrum.

In [15], the authors presented an outage probability (OP) analysis for an IRS-assisted NOMA downlink with linear energy scavenging, yet the derivations were solely applicable to the best- and worst-case scenarios with closed-form OP expressions under Rayleigh fading. The authors of [16] conducted an error performance analysis for an IRS-assisted NOMA downlink but did not optimize system parameters or investigate energy scavenging. Additionally, [16] focused on Rayleigh fading, which is less comprehensive than Nakagami-*m* fading. In [17], the authors explored NOMA in unmanned aerial vehicle systems with multiple IRSs and optimized the system throughput effectively for Rician fading links, neglecting Nakagami-*m* fading and energy harvesting. The study in [18] aimed to minimize the delay and energy for an IRS-assisted NOMA uplink with linear energy harvesting over Rayleigh and Rician fading links, without considering Nakagami-*m* fading or nonlinear energy harvesting, and did not analyze system performance. In [19], the authors designed an IRS-assisted NOMA and analyzed its bit error rate, incorporating Nakagami-*m* fading but ignoring energy harvesting. The authors of [20] analyzed the effective ergodic capacity and OP of IRS-assisted NOMA downlink/uplink, but the analysis was restricted to Rayleigh fading and linear energy harvesting. In [21], the average age of information, sum throughput, and OP of IRS-assisted NOMA with energy harvesting were studied for Nakagami-*m* fading, yet linear energy harvesting was considered, not reflecting real-world ES. For ultra-massive machine type communications, [22] investigated IRS-assisted NOMA downlink with energy harvesting, optimizing the sum rate of all users with FD NOMA communications, while [23] addressed the problem of clustering users and assigning an IRS subject to linear energy scavenging and Rician fading, without conducting a performance analysis. Additionally, works in [15,16,17,18,19,20,21,22,23] did not examine the cognitive radio context.

### 1.2. Motivations and Contributions

In recent years, the advent of IRS has heralded a new era in wireless communication, offering a promising solution to enhance the performance of wireless networks. IRS technology leverages a large array of passive reflecting elements, which can be dynamically adjusted to manipulate electromagnetic waves in a desirable manner, thereby improving signal propagation conditions. This innovative approach has garnered substantial interest in both academia and industry, aiming to develop more efficient and flexible wireless communication systems. A significant body of research has explored the integration of IRS with various wireless communication paradigms. For instance, Wu et al. [24] demonstrated the potential of IRS in enhancing wireless networks through joint active and passive beamforming design. Similarly, Han et al. [25] exploited statistical channel state information (CSI) to optimize the performance of large intelligent surfaces in wireless communications. These studies underscore the capability of IRS to significantly improve signal quality and coverage. Moreover, Huang et al. [26] investigated the energy efficiency benefits of IRS in wireless communication, revealing that IRS can effectively reduce energy consumption while maintaining high performance. Guan et al. [27] further explored the role of artificial noise in IRS-assisted secrecy communications, providing insights into the security enhancements achievable with IRS technology. Comprehensive surveys by Gong et al. [28] and Liu et al. [29] together provide a detailed overview of the principles, challenges, and opportunities associated with IRS, highlighting the transformative potential of this technology. Wu and Zhang [30] discussed the broader vision of smart and reconfigurable environments enabled by IRS, positioning it as a key enabler for future wireless networks. Additionally, Guo et al. [31] focused on the optimization of the weighted sum-rate in IRS-enhanced networks, emphasizing the importance of performance optimization in practical applications. Despite the extensive research on IRS, its application in cognitive radio (CR) systems remains relatively underexplored. CR technology, characterized by its ability to dynamically adapt to the spectrum environment and mitigate interference, can benefit significantly from the integration of IRS. This study aims to bridge this gap by investigating the outage performance of IRS-assisted CR systems. By leveraging IRS, we can potentially achieve better interference management and improved outage performance, thereby enhancing the overall efficiency of CR networks. This study builds upon the foundational work of the aforementioned studies and extends it to the context of CR systems. We compare our results with those presented in previous works, such as [32], demonstrating the superiority of our approach in terms of outage probability and performance optimization. In summary, this manuscript contributes to the growing body of knowledge on IRS by exploring its integration with CR systems, highlighting the benefits and performance improvements achievable through this synergy. The subsequent sections will delve into the system model, performance analysis, and comparative results, providing a comprehensive evaluation of the proposed IRS-assisted CR model.

This study integrates RISs with CR technology, aiming to address the aforementioned gap. The key contributions of this study can be summarized as follows:

- Our proposition involves RIS-assisted CR networks, wherein the base station (BS) communicates signals to two users, referred to as the primary user (PU) and secondary user (SU), via direct and reflected signal paths, respectively. This strategy aligns with the standards of 6G and beyond networks, thereby enhancing practicality and applicability in contemporary network paradigms.
- We develop mathematical formulations for SU within the RIS-assisted CR system. We validate the accuracy and efficacy of these formulations through Monte Carlo simulations.
- We conduct a comprehensive analysis of the performance of RIS-assisted CR systems. Our examination covers various factors such as the influence of SNR, power allocations, the quantity of reflected surfaces, and variations in blocklength. These analyses provide valuable insights that can guide the thoughtful design of RIS-assisted CR systems.

### 1.3. Organization and Notation

***Organization:*** This paper is structured as follows: In Section 2, we present the system model and examine the channel characteristics. Proceeding to Section 3, we conduct an indepth analysis of the outage probability in RIS-assisted CR systems. Section 4 is dedicated to analyzing the EE, while Section 5 presents the EE of the system. In Section 6, we present and discuss the simulation results, followed by the conclusion of this paper in Section 7.

***Notation:*** $\left|.\right|$ denotes the absolute value. $\mathrm{Pr}\left(.\right)$ depicts the probability operator; $E\left\{.\right\}$ is the expectation operator; $\mathrm{diag}\left(.\right)$ represents a diagonal matrix; the superscript ${\left(.\right)}^{T}$ stands for the transpose operator; $\Gamma \left(.\right)$ is the so-called Gamma function; $\gamma \left(.,.\right)$ and $\Gamma \left(.,.\right)$ represent the lower and upper incomplete Gamma functions, respectively. Additionally, the probability density function (PDF) and the cumulative distribution function (CDF) of a random variable *X* are symbolized as $F_{X}\left(.\right)$ and $f_{X}\left(.\right)$, respectively.

## 2. System Model and Channel Characteristics

### System Model

**Figure 1:**
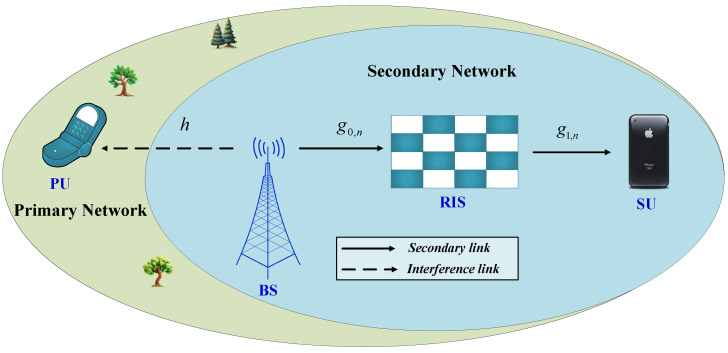
An illustration of RIS-assisted cognitive networks.

Let us examine a downlink system where a RIS supports multiple access in the secondary network (SN). This network comprises a PU, a secondary source functioning as the BS, a secondary RIS featuring *N* reflecting elements, and a SU, depicted in Figure 1. We will make the assumption that each node is outfitted with a single antenna and that there is no direct communication path between the BS and the SU. Let us represent $h\in C^{1\times 1}$ as the channel coefficient from the BS to the PU, $g_{0,n}\in C^{1\times N}$ as the channel vector from the BS to the RIS, and $g_{1,n}\in C^{N\times 1}$ as the channel vector from the RIS to the SU. In our analysis, we utilize a general fading distribution, specifically Nakagami-*m*, for all transmission links. Furthermore, we presume that all channel coefficients associated with the RIS follow an independent and identically distributed (i.i.d.) pattern.

We define $P_{S}$ as the average transmit power from the BS to facilitate transmissions within the SN, aimed at constraining interference to the PU [33].

(1) $$P_{S}\leq \min\left(\frac{I}{{\left|h\right|}^{2}},{\bar{P}}_{S}\right),$$

Here, *I* represents the peak interference temperature power at the PU, while ${\bar{P}}_{S}$ represents the maximum available power of the BS. Let $x_{S}$ denote the transmit signals at the BS, and then it is computed as $\tilde{x}=\sqrt{P_{S}}x_{S}$. Here, we make the assumption that the transmitted signals are normalized, indicating that $E\left\{{\left|x_{S}\right|}^{2}\right\}=1$, where $E\left\{.\right\}$ denotes the expectation operator. The signal received at the SD from the BS to the RIS and from the RIS to the SU is then given by:

(2) $${\bar{y}}_{SD}=g_{1}^{T}\Phi g_{0}\tilde{x}+n_{SD},$$

In this expression, $\Phi =\mathrm{diag}\left(\beta_{1}e^{j\theta_{1}},\ldots ,\beta_{n}e^{j\theta_{n}},\ldots ,\beta_{N}e^{j\theta_{N}}\right)$ represents a diagonal matrix where $0<\beta_{n}\leq 1$ for $n\in \left\{1,\ldots ,N\right\}$, denoting the amplitude reflection coefficient of the *n*-th reflecting element. Additionally, $\theta_{n}\in \left[0,2\pi \right)$ denotes the phase shift of the *n*-th reflecting element. Here, $e^{\left(.\right)}=\exp\left(.\right)$ is the exponential function, $g_{1}={\left[g_{1,1},\ldots ,g_{1,n},\ldots ,g_{1,N}\right]}^{T}$, $g_{0}={\left[g_{0,1},\ldots ,g_{0,n},\ldots ,g_{0,N}\right]}^{T}$, and ${\left(.\right)}^{T}$ denotes the transpose operation. Let ${\bar{g}}_{0,n}=g_{0,n}e^{j\varphi_{n}}$ and ${\bar{g}}_{1,n}=g_{1,n}e^{j\phi_{n}}$ denote the channel coefficients from the BS to the RIS and from the RIS to the SU, respectively, where $\varphi_{n},\phi_{n}\in \left[0,2\pi \right)$ is the phase shift of $g_{0,n}$ and $g_{1,n}$. $n_{SD}$ depicts the additive white Gaussian noise (AWGN) with zero mean and variance $\sigma_{SD}^{2}$. The received signals in (1) can thus be reformulated as:

(3) $$\begin{array}{cc} {\bar{y}}_{SU}= & \sqrt{P_{S}}x_{S}\sum_{n=1}^{N}{\bar{g}}_{0,n}{\bar{g}}_{1,n}e^{j\theta_{n}}+n_{SD}\\ = & \sqrt{P_{S}}x_{S}\sum_{n=1}^{N}g_{0,n}g_{1,n}e^{j\zeta_{n}}+n_{SD}, \end{array}$$

Here, $\zeta_{n}=\theta_{n}+\phi_{n}+\varphi_{n}$. The equivalent end-to-end (E2E) signal-to-noise (SNR) at the SU is then expressed as:

(4) $${\bar{\gamma }}_{SU}=\rho_{S}{\left|\sum_{n=1}^{N}g_{0,n}g_{1,n}e^{j\zeta_{n}}\right|}^{2},$$

Here, ρS=PSPSσSU2σSU2 denotes the average transmit SNR. In this scenario, we presume a high phase-shift resolution and perfect Channel State Information (CSI) at the RIS. Channel estimation can be achieved by employing the methodology outlined in [34]. Subsequently, the optimal phase-shift design is utilized to maximize the SNR at the destination [35]. Specifically, let $\theta_{n}^{✲}$ denote the optimal phase-shift of the *n*-th element of the RIS. Its value is then determined by:

(5) $$\theta_{n}^{✲}=-\phi_{n}-\varphi_{n},\forall n.$$

The optimal instantaneous SNR at the SU can be rewritten as:

(6) $${\bar{\gamma }}_{SU}^{otp}=\rho_{S}{\left|\sum_{n=1}^{N}g_{0,n}g_{1,n}\right|}^{2}=\rho_{S}{\left|A\right|}^{2},$$

where $A\overset{\Delta }{\;=}\sum_{n=1}^{N}g_{0,n}g_{1,n}$. Next, we introduce some distributions that will be utilized in the performance analysis. Let *X* be a random variable (RV) following a Nakagami-*m* distribution, characterized by its PDF and CDF, parameterized by *m* and Ω, as provided by [36]:

(7a) $$\begin{array}{cc} \; & f_{X}\left(x;m,\Omega \right)=\frac{2m^{m}}{\Gamma \left(m\right)\Omega^{m}}x^{2m-1}e^{-\frac{m}{\Omega }x^{2}}, \end{array}$$

(7b) $$\begin{array}{cc} \; & F_{X}\left(x;m,\Omega \right)=\frac{\gamma \left(m,\frac{m}{\Omega }x^{2}\right)}{\Gamma \left(m\right)}, \end{array}$$

Here, m>0 serves as the shape parameter, indicating the severity of fading, while Ω>0 stands as the spread parameter of the distribution. We employ an alternative notation to represent a Nakagami-*m* random variable: $X∼\mathrm{Nakagami}\;\left(m,\Omega \right)$. It is worth noting that Ω represents the mean square value of *X*, denoted as $E\left\{X^{2}\right\}$ [37], which equates to the average channel (power) gain. The distribution of the magnitude of each individual channel is articulated as follows: $h∼\mathrm{Nakagami}\left(m_{h},\Omega_{h}\right)$, $g_{0,n}∼\mathrm{Nakagami}\;\left(m_{0},\Omega_{0}\right)$, and $g_{1,n}∼\mathrm{Nakagami}\;\left(m_{1},\Omega_{1}\right)$, where n=1,…,N. Let *Y* be a RV following a Gamma distribution, characterized by its PDF and CDF, parameterized by χ and δ, respectively, as provided by [36]:

(8a) $$\begin{array}{cc} \; & f_{Y}\left(y;\chi ,\delta \right)=\frac{\delta^{\chi }}{\Gamma \left(\chi \right)}y^{\chi -1}e^{-\delta y}, \end{array}$$

(8b) $$\begin{array}{cc} \; & F_{Y}\left(y;\chi ,\delta \right)=\frac{\gamma \left(\chi ,\delta y\right)}{\Gamma \left(\chi \right)}, \end{array}$$

Here, χ>0 serves as the shape parameter, while $\delta_{A}>0$ stands as the rate parameter of the distribution. Subsequently, we adopt the following representation to denote a Gamma RV: $Y∼\mathrm{Nakagami}\;\left(\chi_{A},\delta \right)$. Utilizing the proposed distribution estimation framework, we demonstrate that the true distribution of A is accurately approximated by the Gamma distribution. The true distribution of A can be approximated by the Gamma distribution, characterized by two parameters $\chi_{A}$ and $\delta_{A}$, denoted as $A∼Gamma\;\left(\chi_{A},\delta_{A}\right)$. The estimators of $\chi_{A}$ and $\delta_{A}$ can be expressed as cited in [38].

(9) $$\chi_{A}=\frac{{\left(E\left\{A\right\}\right)}^{2}}{\mathrm{Var}\left\{A\right\}}=\frac{{\left[\mu_{A}\left(1\right)\right]}^{2}}{\mu_{A}\left(2\right)-{\left[\mu_{A}\left(1\right)\right]}^{2}},$$

and

(10) $$\delta_{A}=\frac{E\left\{A\right\}}{\mathrm{Var}\left\{A\right\}}=\frac{\mu_{A}\left(1\right)}{\mu_{A}\left(2\right)-{\left[\mu_{A}\left(1\right)\right]}^{2}},$$

respectively, where $\mu_{A}\left(1\right)$ and $\mu_{A}\left(2\right)$ are presented in [38]. Therefore, the approximate PDF and CDF of A, denoted as $f_{Y}\left(y;\alpha ,\beta \right)$ and $F_{Y}\left(y;\alpha ,\beta \right)$, respectively, can be expressed using Equations (8a) and (8b) as provided. With the PDF of A determined, we proceed to derive the *k*-th moment of A as follows:

(11) $$\mu_{A}\left(k\right)=\frac{\Gamma \left(m_{0}+0.5k\right)\Gamma \left(m_{1}+0.5k\right)}{\Gamma \left(m_{0}\right)\Gamma \left(m_{1}\right)}{\left(\frac{\Omega_{0}\Omega_{1}}{m_{0}m_{1}}\right)}^{\frac{k}{2}}.$$

Indeed, determining the statistical characteristics of the Gamma distribution, specifically $\mu_{A}\;\left(1\right)$ and $\mu_{A}\;\left(2\right)$, can pose a challenge. Additionally, acknowledging that for arbitrary *X* and *Y*, where $Y=X^{2}$, we have $F_{Y}\left(y\right)=F_{X}\left(\sqrt{y}\right)$ and $f_{Y}\left(y\right)=\frac{1}{2\sqrt{y}}f_{X}\left(\sqrt{y}\right)$, the PDF and CDF of $A^{2}$ can be obtained as:

(12a) $$\begin{array}{cc} \; & f_{A^{2}}\left(x\right)\approx \frac{\delta_{A}^{\chi_{A}}}{2\sqrt{x}\Gamma \left(\chi_{A}\right)}x^{\frac{\chi_{A}-1}{2}}e^{-\delta_{A}\sqrt{x}}, \end{array}$$

(12b) $$\begin{array}{cc} \; & F_{A^{2}}\left(x\right)\approx \frac{\gamma \left(\chi_{A},\delta_{A}\sqrt{y}\right)}{\Gamma \left(\chi_{A}\right)}, \end{array}$$

Moreover, Nakagami-distributed RVs of ${\left|h\right|}^{2}$ exhibit exponential distributions, as indicated in [39]

(13a) $$\begin{array}{cc} \; & f_{{\left|h\right|}^{2}}\left(x\right)=\frac{\mu^{m_{h}}e^{-\mu x}x^{m_{h}-1}}{\Gamma \left(m_{h}\right)}, \end{array}$$

(13b) $$\begin{array}{cc} \; & F_{{\left|h\right|}^{2}}\left(x\right)=1-e^{-\mu x}\sum_{s=0}^{m_{h}-1}\frac{\mu^{s}x^{s}}{s!}, \end{array}$$

Here, $\Gamma \left(x\right)=\left(x-1\right)!$ represents the Gamma function, and $\mu =\frac{m_{h}}{\Omega_{h}}$, where $\Omega_{h}$ and $m_{h}$ denote the mean and integer fading factor, respectively.

## 3. Outage Probability Analysis

### 3.1. Exact Calculation of OP

In a recent study [40], the performance of devices in both SU and PU has been considered. However, we prioritize the examination of device performance at SU. It is anticipated that devices operating in the SU face limited performance due to the power constraint of the secondary transmitter in Equation (2). As the primary performance evaluation metric, we employ the OP, which represents the probability of the corresponding SNR falling below a predefined threshold λ, denoted as $P_{out}=\mathrm{Pr}\left(Z<\lambda \right)=F_{Z}\left(\lambda \right)$ [41].

The OP of the SU is calculated as follows:

(14) $$\begin{array}{cc} O_{SU}= & \mathrm{Pr}\left({\bar{\gamma }}_{SU}^{otp}<\gamma_{th}\right)\\ = & 1-\mathrm{Pr}\left({\bar{\gamma }}_{SU}^{otp}>\gamma_{th}\right), \end{array}$$

Here, $\gamma_{th}=2^{R}-1$ represents the target SNR at the SU, where *R* denotes the pre-data transmission rate of the device.

Substituting the expression for $O_{SU}$ from Equation (6) into Equation (14), we obtain:

(15) $$O_{SU}=1-\mathrm{Pr}\left({\left|A\right|}^{2}>\frac{\gamma_{th}}{\rho_{S}}\right).$$

We note that the expression for $\rho_{S}$, given by $\rho_{S}=\min\left({\bar{\rho }}_{S},\frac{\rho_{I}}{{\left|h\right|}^{2}}\right)$, (15), is calculated as follows:

(16) $$O_{SD}=1-\left(B_{1}+B_{2}\right),$$

where $B_{1}=\mathrm{Pr}\left({\bar{\rho }}_{S}{\left|A\right|}^{2}>\gamma_{th},{\left|h\right|}^{2}<\frac{\rho_{I}}{{\bar{\rho }}_{S}}\right)$ and $B_{2}=\mathrm{Pr}\left(\rho_{I}{\left|A\right|}^{2}>\gamma_{th}{\left|h\right|}^{2},{\left|h\right|}^{2}>\frac{\rho_{I}}{{\bar{\rho }}_{S}}\right)$, where ρ¯S=P¯AP¯AN0N0 represents the average SNR at the BS and ρI=IIN0N0 depicts the average SNR of interference at the PU.

**Proposition 1.**  *The closed-form expression of OP at the SU is expressed as Equation (17),*

(17) $$\begin{array}{c} O_{SU}=1-\frac{\gamma \left(m_{h},\frac{\mu \rho_{I}}{{\bar{\rho }}_{S}}\right)\Gamma \left(\chi_{A},\delta_{A}\sqrt{\frac{\gamma_{th}}{{\bar{\rho }}_{S}}}\right)}{\Gamma \left(\chi_{A}\right)\Gamma \left(m_{h}\right)}-\\ \;\;\frac{1}{\Gamma \left(m_{h}\right)}\left[\Gamma \left(m_{h},\frac{\rho_{I}}{{\bar{\rho }}_{S}}\mu \right)-\sum_{q=0}^{\infty }\frac{{\left(-1\right)}^{q}\delta_{A}^{\chi_{A}+q}\gamma_{th}^{\frac{\chi_{A}+q}{2}}}{q!\Gamma \left(\chi_{A}\right)\left(\chi_{A}+q\right){\left(\rho_{I}\mu \right)}^{\frac{\chi_{A}+q}{2}}}\Gamma \left(\frac{\chi_{A}+q+2m_{h}}{2},\frac{\rho_{I}\mu }{{\bar{\rho }}_{S}}\right)\right]. \end{array}$$

**Proof.**  The proof is provided in Appendix A.    □

### 3.2. Asymptotic Calculation of Key Performance Indicators

Since deriving closed-form expressions may not provide significant insight, we opt to analyze asymptotic expressions to gain further intuition.

As the average SNR ${\bar{\rho }}_{S}$ approaches infinity, we observe that $B_{1}\approx 0$ and $\frac{\rho_{I}}{{\bar{\rho }}_{S}}\approx 0$. In this limit, the asymptotic expression for $O_{SU}^{\infty }$ is calculated as:

(18) $$O_{SU}^{\infty }=1-\mathrm{Pr}\left({\left|A\right|}^{2}>\frac{\gamma_{th}}{\rho_{I}}{\left|h\right|}^{2}\right).$$

We can express Equation (18) at the SU as follows:

(19) $$\begin{array}{cc} O_{SU}^{\infty }= & 1-\int_{0}^{\infty }f_{{\left|h\right|}^{2}}\left(x\right)\left[1-F_{{\left|A\right|}^{2}}\left(\frac{\gamma_{th}}{\rho_{I}}x\right)\right]dx\\ = & 1-\int_{0}^{\infty }f_{{\left|h\right|}^{2}}\left(x\right)dx+\int_{0}^{\infty }f_{{\left|h\right|}^{2}}\left(x\right)F_{{\left|A\right|}^{2}}\left(\frac{\gamma_{th}}{\rho_{I}}x\right)dx. \end{array}$$

By substituting Equations (13a) and (12b) into Equation (19) and performing several steps, we derive the asymptotic expression at the SU as follows:

(20) $$\begin{array}{cc} O_{SU}^{\infty }= & 1-\frac{\mu^{m_{h}}}{\Gamma \left(m_{h}\right)}\int_{0}^{\infty }e^{-\mu x}x^{m_{h}-1}dx+\frac{\mu^{m_{h}}}{\Gamma \left(m_{h}\right)\Gamma \left(\chi_{A}\right)}\\ \; & \times \sum_{q=0}^{\infty }\frac{{\left(-1\right)}^{q}\delta_{A}^{\chi_{A}+q}\gamma_{th}^{\frac{\chi_{A}+q}{2}}}{q!\left(\chi_{A}+q\right)\rho_{I}^{\frac{\chi_{A}+q}{2}}}\int_{0}^{\infty }e^{-\mu x}x^{\frac{\chi_{A}+q+2m_{h}-2}{2}}dx. \end{array}$$

Utilizing this, we express Equation (20) as follows:

(21) $$O_{SU}^{\infty }=\sum_{q=0}^{\infty }\frac{{\left(-1\right)}^{q}\delta_{A}^{\chi_{A}+q}\gamma_{th}^{\frac{\chi_{A}+q}{2}}\Gamma \left(\frac{\chi_{A}+q+2m_{h}}{2}\right)}{q!\Gamma \left(m_{h}\right)\Gamma \left(\chi_{A}\right)\left(\chi_{A}+q\right)\rho_{I}^{\frac{\chi_{A}+q}{2}}\mu^{\frac{\chi_{A}+q}{2}}}.$$

**Remark 1.**  *Based on the definition of the diversity order, denoted as $\bar{d}=-\lim_{{\bar{\rho }}_{S}\to \infty }\frac{\log\left(O_{SU}^{\infty }\right)}{\log\left({\bar{\rho }}_{S}\right)}$, as ${\bar{\rho }}_{S}$ approaches infinity, the diversity order of 0 is attained. Therefore, we can anticipate the existence of an error floor at a high transmit SNR at the BS, similar to the findings in [42].*

### 3.3. Throughput Analysis

In this section, we perform an optimal analysis of the throughput at the SU, denoted by $\tau_{SU}^{✲}$, in the examined downlink scenario of RIS-assisted cognitive systems. More specifically, we introduce a method for computing the optimal value of $R^{✲}$, leading to the system’s optimum throughput.

Building upon outage performance analysis, we extend our examination to include the metric of throughput in delay-limited transmission mode. Throughput represents the system’s capacity when a fixed data rate is mandated. The throughput at these key nodes can be obtained as follows:

(22) $$\begin{array}{c} \tau_{SU}=\left(1-O_{SU}\right)R. \end{array}$$

The optimal points of throughput as the target rates of *R* vary are expressed as:

(23) $$\tau_{SU}^{✲}=\arg\max\left\{\tau_{SU}\left(R\right)\right\}.$$

Using Algorithm 1, the optimal throughput values may be found correctly. We intend to validate such a method using Matlab (version 2019a) as follows:
**Algorithm 1:** The algorithm of finding the optimal throughput coefficient $\tau_{SU}^{✲}$.
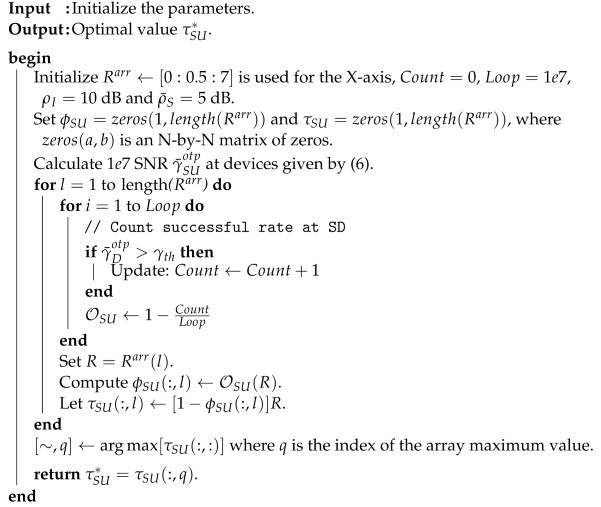


## 4. Ergodic Rate Analysis

When the device rate is dictated by channel conditions, the ergodic rate (ER) serves as a useful indicator for performance assessment. In contrast to [43], our aim is to derive an approximate expression for ER. Essentially, ER is defined as the long-term average achievable data rate obtained without considering any delay constraints. We proceed to investigate the ER of the system. The achievable rate of the considered system at the SU is given by [44].

(24) $$C_{SU}=E\left\{\log_{2}\left(1+{\bar{\gamma }}_{SD}^{otp}\right)\right\}.$$

The ER of the SU can be obtained using Proposition 2.

**Proposition 2.**  *The closed-form approximate expression for the ER at the SU is provided by:*

(25) $$\begin{array}{cc} C_{SU}\approx & \frac{\pi^{2}}{8U\ln2}\left[\sum_{u=1}^{U}\frac{\Delta_{1}\sqrt{1-\xi_{u}^{2}}\Xi \left(\xi_{u}\right)}{1+g\left(\xi_{u}\right)}\right.\\ \; & \times \Gamma \left(\chi_{A},\delta_{A}\sqrt{{\bar{\rho }}_{S}^{-1}g\left(\xi_{u}\right)}\right)+\sum_{u=1}^{U}\frac{\sqrt{1-\xi_{u}^{2}}}{1+g\left(\xi_{u}\right)}\\ \; & \left.\times \Xi \left(\xi_{u}\right)\left(I_{1}-\sum_{q=0}^{\infty }\Delta_{2}g{\left(\xi_{u}\right)}^{\frac{\chi_{A}+q}{2}}\right)\right], \end{array}$$

*where $V_{1}=\frac{\gamma \left(m_{h},\frac{\mu \rho_{I}}{{\bar{\rho }}_{S}}\right)\Gamma \left(\chi_{A},\delta_{A}\sqrt{\frac{x}{{\bar{\rho }}_{S}}}\right)}{\Gamma \left(\chi_{A}\right)\Gamma \left(m_{h}\right)}$ and $V_{2}=I_{1}-\sum_{q=0}^{\infty }\frac{{\left(-1\right)}^{q}\delta_{A}^{\chi_{A}+q}x^{\frac{\chi_{A}+q}{2}}}{q!\left(\chi_{A}+q\right)\rho_{I}^{\frac{\chi_{A}+q}{2}}}I_{2}$ in which $I_{1}=\frac{1}{\Gamma \left(m_{h}\right)}\Gamma \left(m_{h},\frac{\rho_{I}}{{\bar{\rho }}_{S}}\mu \right)$, $I_{2}=\frac{\Gamma \left(\frac{\chi_{A}+q+2m_{h}}{2},\frac{\rho_{I}\mu }{{\bar{\rho }}_{S}}\right)}{\Gamma \left(m_{h}\right)\Gamma \left(\chi_{A}\right)\mu^{\frac{\chi_{A}+q-2}{2}}}$, $\Delta_{1}=\frac{\gamma \left(m_{h},\frac{\mu \rho_{I}}{{\bar{\rho }}_{S}}\right)}{\Gamma \left(\chi_{A}\right)\Gamma \left(m_{h}\right)}$, $\Delta_{2}=\frac{{\left(-1\right)}^{q}\delta_{A}^{\chi_{A}+q}}{q!\left(\chi_{A}+q\right)\rho_{I}^{\frac{\chi_{A}+q}{2}}}I_{2}$, $g\left(t\right)=\tan\left(\frac{\pi \left(t+1\right)}{4}\right)$, $\Xi \left(t\right)=\sec^{2}\left(\frac{\pi }{4}\left(t+1\right)\right)$ and sec2x=11cos2xcos2x.*

**Proof.**  See Appendix B. □

## 5. Energy Efficiency

Following the determination of throughput in both delay-limited transmission and delay-tolerant scenarios, it becomes imperative to delve deeper into studying the system’s energy efficiency (EE) within RIS-assisted cognitive networks. The coefficient of energy efficiency is formulated as per (Equation (29) in [33]).

(26) $$\eta_{EE}=\frac{\mathrm{Total}\;\;\mathrm{data}\;\;\mathrm{rate}}{\begin{array}{c} \mathrm{Total}\;\;\mathrm{energy}\;\;\mathrm{consumption} \end{array}}.$$

Hence, we can express the EE values for RIS-assisted cognitive network systems as follows:

(27a) $$\begin{array}{cc} \; & \eta_{O_{SU}}^{EE}=\frac{\tau_{SU}}{TP_{S}}, \end{array}$$

(27b) $$\begin{array}{cc} \; & \eta_{C_{SU}}^{EE}=\frac{C_{SU}}{TP_{S}}, \end{array}$$

Here, *T* represents the transmission time allocated for the entire communication process.

## 6. Numerical Results

This section shows the analytical results of the proposed system through simulations. Here, we set $m=m_{h}=m_{0}=m_{1}$ and numerically simulate several theoretical results to demonstrate the outage performance. The other main parameters are summarized in Table 1. Additionally, the Gaussian–Chebyshev parameter is chosen as U=100 to achieve a close approximation.

**Figure 2:**
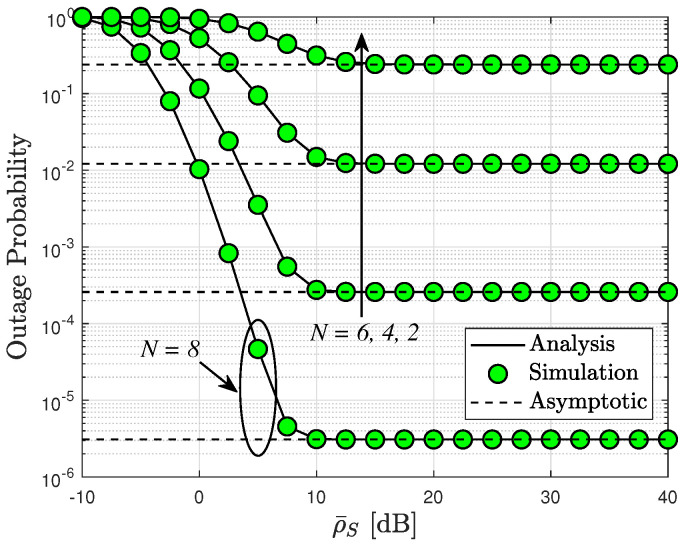
Outage probability versus ${\bar{\rho }}_{S}$ [dB] with different $N=\left\{2,4,6,8\right\}$ and m=2.

Similar observations are applicable for different numbers of RIS elements as depicted in Figure 2. Notably, the outage performance of the SU reaches its lowest value for N=8. Furthermore, it is evident that performance improvements can be achieved by adjusting the number of RIS elements, rendering the outage performance suitable for continuous operation of this system.

**Figure 3:**
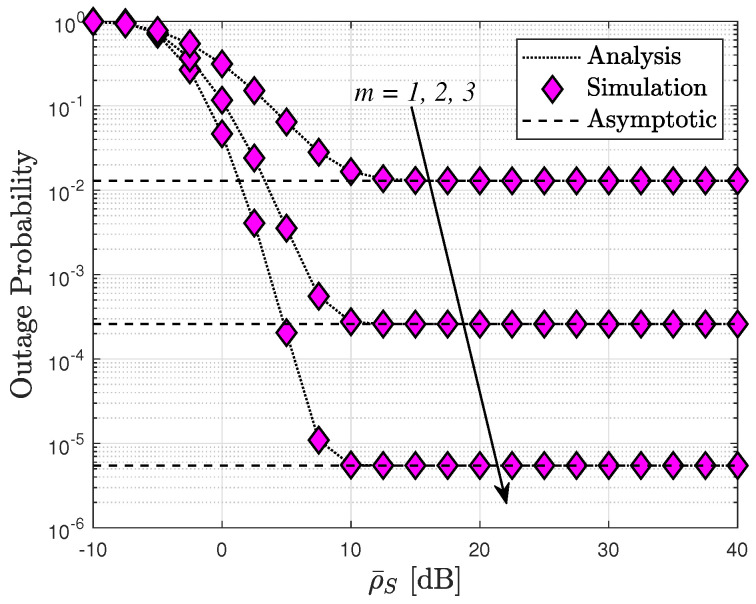
Comparison of outage probability with different *m* fading parameters, with N=6.

Figure 3 illustrates how the increase in transmit power at the BS affects the performance of RIS-assisted cognitive network systems in terms of the outage probability while keeping the number of RIS elements fixed. We aim to assess the influence of channel severity on system performance, considering values of m=1,2,3. Notably, the lowest OP of the SU is attained with a fading parameter of m=3. Furthermore, for large SNR values, an error floor becomes evident, aligning with the asymptotic analysis presented in (21).

**Figure 4:**
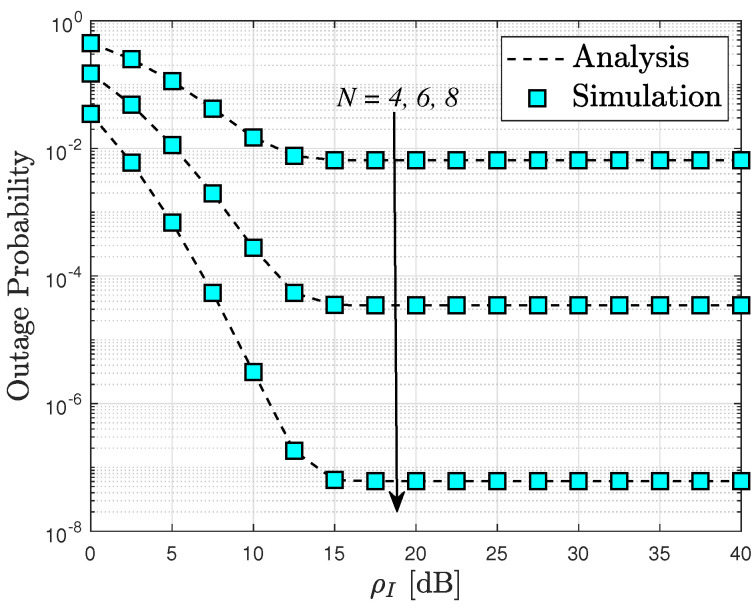
Outage probability versus the maximum available transmit power of the secondary source, with ${\bar{\rho }}_{S}=10$ dB and m=2.

Figure 4 depicts the OP of the RIS-assisted cognitive network systems plotted against $\rho_{I}$. The outage probability is inherently influenced by the interference power, which directly impacts the transmit SINR of the secondary source, as illustrated in this graph. Consequently, the patterns of outage probability observed are comparable to those seen in Figure 2. Moreover, the intuitive observation of saturated curves of the outage probability, as reported in Figure 2, Figure 3 and Figure 4, corroborates the diversity order “0” as mentioned in Remark 1. This phenomenon can be attributed to the fact that such an OP cannot be further improved at high SNR values as it becomes dependent on other system parameters.

**Figure 5:**
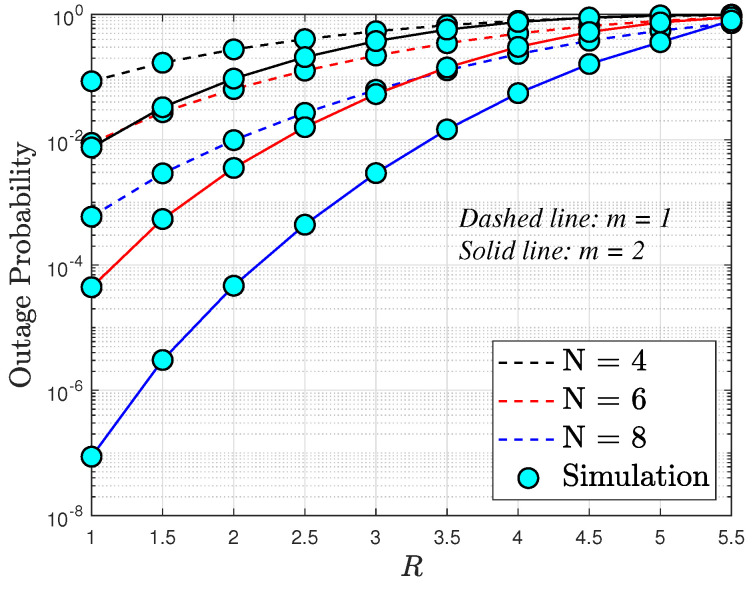
Outage probability versus *R*, with ${\bar{\rho }}_{S}=5$ dB and $\rho_{I}=10$ dB.

In Figure 5, we examine the OP as a function of the targeted data rates *R*, considering different numbers of elements in the RIS N=4,6,8, and transmit SNR $\bar{\rho }=5$ (dB). Once more, we observe that increasing the number of RIS elements leads to enhanced throughput. Notably, the best outage performance is achieved at lower values of *R* rates. It is noteworthy that for downlink RIS-assisted cognitive network systems, Figure 2, Figure 3 and Figure 4 underscore the significant contribution of RIS elements to the OP.

**Figure 6:**
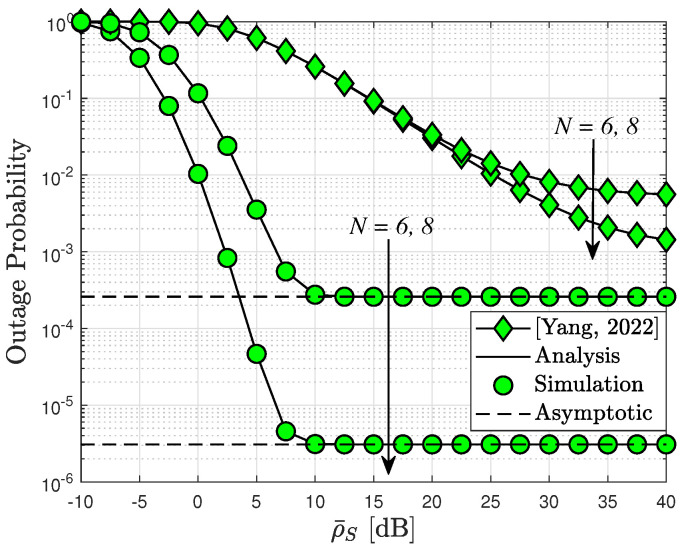
A comparison of the results presented in [32] regarding outage probability, with the parameters N=6,8, m=2, $\rho_{I}=10$ dB, and R=2.

In Figure 6, we present the outage probability versus ${\bar{\rho }}_{S}$ for different values of *N*. It has also been observed that the RIS-assisted CR system achieves a better outage performance than [32]. This is because a higher transmit power of BS can be achieved by eliminating the interference at PU. As can be seen, our model outperforms [32] completely even when increasing the number of elements *N* and transmit power.

**Figure 7:**
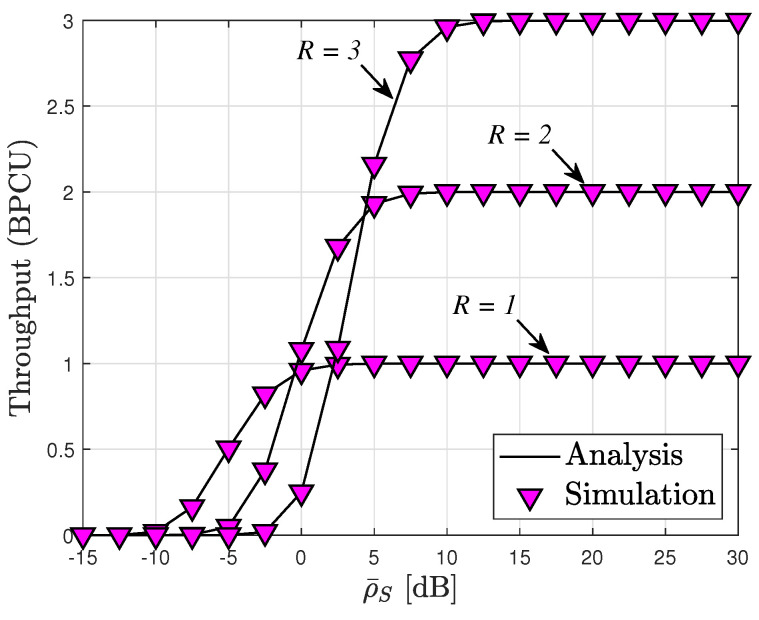
Throughput versus transmit SNR at BS ${\bar{\rho }}_{S}$ with N=6, m=3 and $\rho_{I}=15$ [dB].

From the analysis of the OP metrics, it is evident that the throughput depicted in Figure 7 experiences a notable increase as ${\bar{\rho }}_{S}$ rises from 5 to 30. Interestingly, beyond a certain threshold of ${\bar{\rho }}_{S}$ (>15), the throughput remains unchanged. This phenomenon indicates a saturation point in throughput attainment. In the high $\rho_{S}$ region, the throughput exhibits a ceiling value, consistent with our theoretical analysis. These observations align with the expression derived in (22).

**Figure 8:**
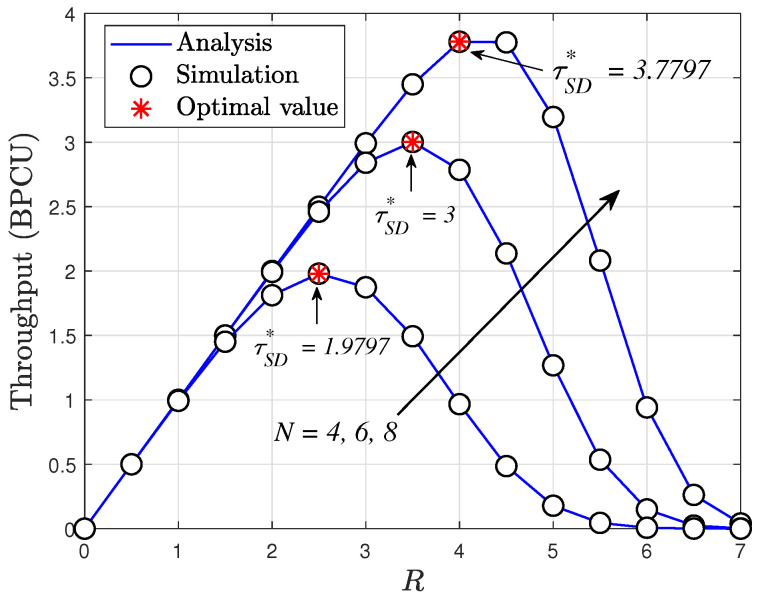
Throughput versus *R* with m=2, ${\bar{\rho }}_{S}=5$ [dB] and $\rho_{I}=10$ [dB].

Figure 8 illustrates the throughput performance of the RIS-assisted cognitive system. It is evident from the plot that the optimal points of throughput vary with different target rates, *R*. This observation is based on the OP, as cases of the OP depend on target rates. In this figure, there exists a specific value of target rates that leads to the highest throughput. For instance, the maximum throughput of the SU occurs at R=4 when N=8. These serve as guidelines to determine the quality of the data rate and throughput for the considered system.

**Figure 9:**
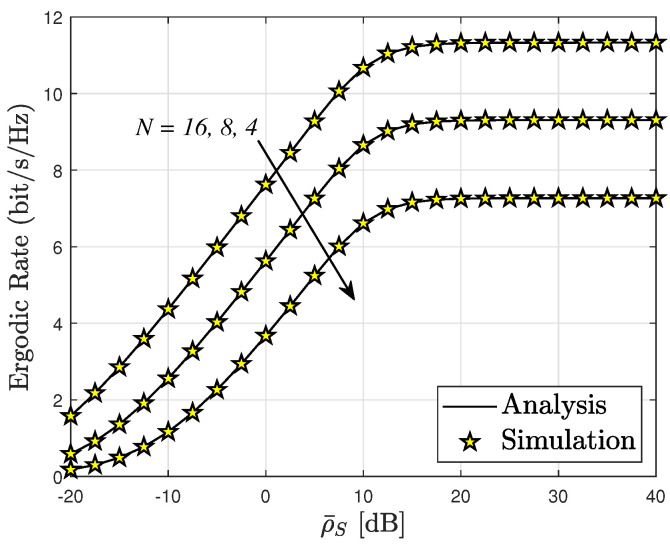
Ergodic rate versus ${\bar{\rho }}_{S}$, with m=2.

Figure 9 illustrates that the ergodic rate of the SU can be enhanced in the high-SNR regime, ${\bar{\rho }}_{S}$, resulting in more reliable transmission. Specifically, Figure 8 presents the ergodic rate performance, where the SU with N=16 exhibits the highest ergodic rate among the three cases examined. The ER of the system experiences a significant increase as ${\bar{\rho }}_{S}$ is augmented from −20 to 40 [dB]. However, beyond a certain threshold (${\bar{\rho }}_{S}>20$ [dB]), the ergodic rate encounters an upper constraint, similarly to the situation observed for the OP.

**Figure 10:**
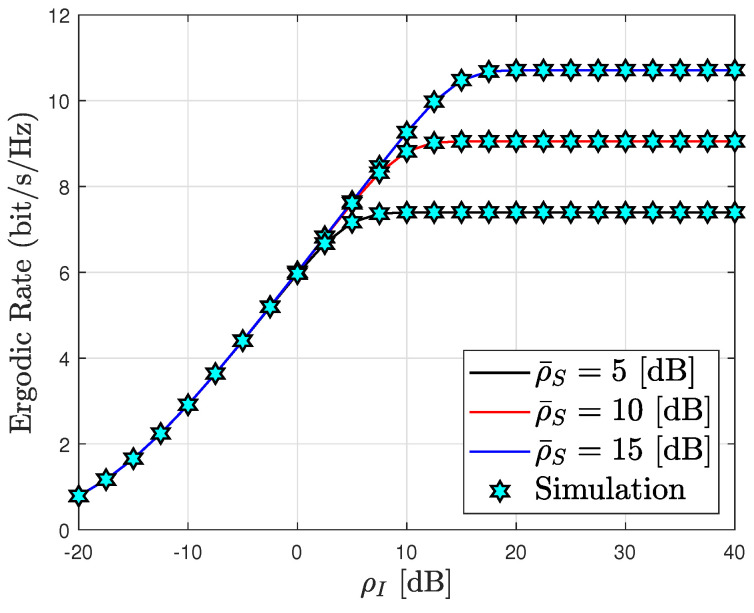
Ergodic rate versus $\rho_{I}$, with m=2 and N=8.

In a similar vein to the preceding observation, Figure 10 demonstrates the effect of interference $\rho_{I}$ on the ER. As depicted, there is a subtle variation in the ergodic rate as $\rho_{I}$ ranges from −20 to 40 dB. This phenomenon can be attributed to the primary factor influencing the ER, which is the transmitted SNR at the source. Consequently, for the secondary network to operate effectively, it is crucial to achieve reasonable levels of transmit SNR and power splitting factors.

**Figure 11:**
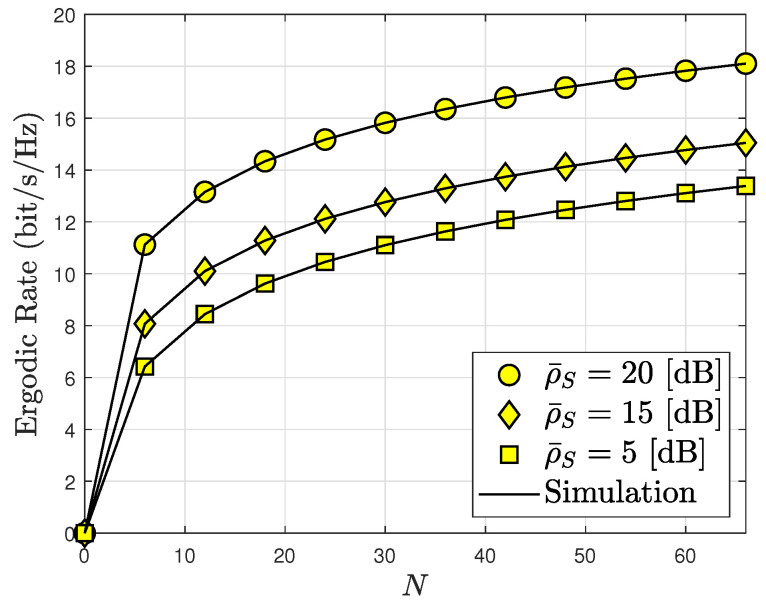
The number of meta-surface influences ergodic capacity, with m=2 and $\rho_{I}=20$ [dB].

Figure 11 shows that increasing the number of metasurfaces *N* on the RIS improves its ergodic capacity. It is evident that the ergodic capacity increases extremely quickly when *N* varies from 0 to 60. After this time, the ergodic capacity only increases marginally. The RIS-assisted system’s ergodic capacity performance for the destination is compared with a set of SNR levels at the BS $\left({\bar{\rho }}_{S}=5,15,20\right)$. Increasing ${\bar{\rho }}_{S}$ and *N* improves the system’s ergodic capacity at low SNR levels. As a result, the creation of several metasurfaces *N* is unnecessary.

**Figure 12:**
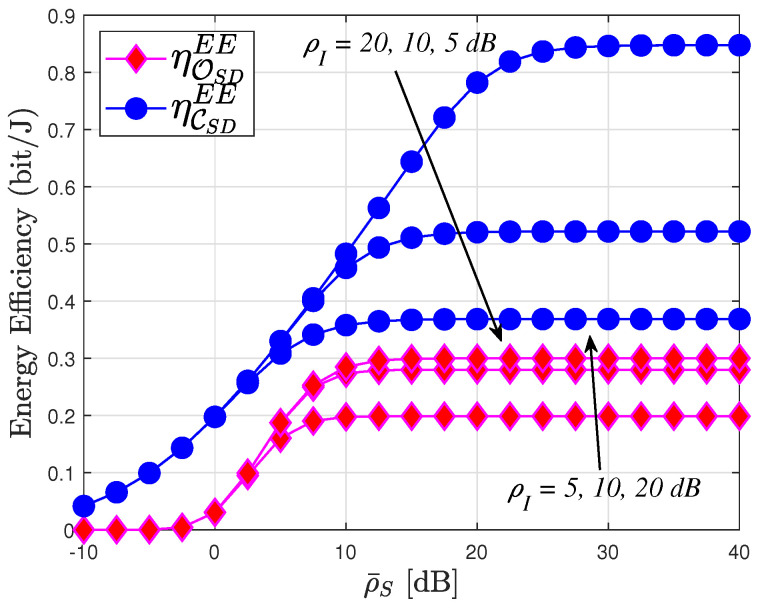
System energy efficiency transmit SNR at the BS, with T=1, $P_{S}=10$ W, R=3, N=4 and m=2.

Figure 12 compares the system EE to the SNR at the source in two modes, namely delay-limited transmission and delay-tolerant transmission, for three relevant instances with interference power levels: $\rho_{I}=20,10,5$ dB. Notably, the system EE in delay-limited transmission mode consistently falls below that in delay-tolerant transmission mode across all three scenarios. Specifically, $\rho_{I}=20$ exhibits the highest value of system EE among the three instances. However, it is important to note that the system’s EE is constrained at high transmit SNR levels. This limitation arises because the system’s EE is determined by both OP and ER, which correspond to delay-limited and delay-tolerant transmission modes, respectively, while both OP and ER performances reach saturation at high SNR levels. This phenomenon aligns with observations presented in Figure 2, Figure 3 and Figure 4, Figure 6, Figure 8 and Figure 9.

## 7. Conclusions

In this paper, we present a thorough analysis of the proposed RIS-assisted CRS under practical operational conditions, taking Nakagami-*m* fading into account. The analysis provides detailed insights into RIS-assisted CRS, enabling efficient performance evaluation across various key parameters. Additionally, it includes a quick comparison of RIS-assisted CRS performance. The study illustrates that the performance of RIS-assisted CRS is significantly affected by factors such as the number of reflectors, fading severity, and $\rho_{I}$. It also demonstrates that careful selection of parameters such as *R*, *N*, *m*, and $\rho_{I}$ can prevent a complete outage, highlighting the system’s robustness. Furthermore, the analysis suggests that optimizing the configuration of *R* can lead to improved system performance. Moreover, the study indicates that RIS-assisted CRS outperforms its reference scheme, further validating its efficacy.

## Figures and Tables

**Table 1:** Main parameters for our simulations [44,45].

Parameters	Notation	Values
Monte Carlo simulations	−	$10^{7}$ iterations
Target rate	*R*	2 (bps/Hz)
Transmit power to noise ratio at BS	${\bar{\rho }}_{S}$	−10 to 30 (dB)
The interference constraint at PU	$\rho_{I}$	10 (dB)
The fading parameter	*m*	2
Transmission time	*T*	1
Mean channel gains	$\Omega_{h}$	1
	$\Omega_{0}$	1
	$\Omega_{1}$	1

## Data Availability

Data are contained within the article.

## Appendix A. Proof of Proposition 1

Based on Equation (16), aided by Equation (12a) and the PDF of ${\left|h\right|}^{2}$, $B_{1}$ can be further computed as follows:

(A1) $$\begin{array}{cc} B_{1}= & \mathrm{Pr}\left({\bar{\rho }}_{S}{\left|A\right|}^{2}>\gamma_{th},{\left|h\right|}^{2}<\frac{\rho_{I}}{{\bar{\rho }}_{S}}\right)\\ = & \int_{\frac{\gamma_{th}}{{\bar{\rho }}_{S}}}^{\infty }f_{{\left|A\right|}^{2}}\left(x\right)\int_{0}^{\frac{\rho_{I}}{{\bar{\rho }}_{S}}}f_{{\left|h\right|}^{2}}\left(y\right)dxdy\\ = & \frac{\delta_{A}^{\chi_{A}}\mu^{m_{h}}}{2\Gamma \left(\chi_{A}\right)\Gamma \left(m_{h}\right)}\int_{0}^{\frac{\gamma_{th}}{{\bar{\rho }}_{S}}}x^{\frac{\chi_{A}-2}{2}}e^{-\delta_{A}\sqrt{x}}\int_{0}^{\frac{\rho_{I}}{{\bar{\rho }}_{S}}}e^{-\mu y}y^{m_{h}-1}dxdy. \end{array}$$

Next, $B_{1}$ can be obtained by using (Equations (3.351.1) and (3.351.2) in [46]), and it is tantamount to

(A2) $$B_{1}=\frac{\gamma \left(m_{h},\frac{\mu \rho_{I}}{{\bar{\rho }}_{S}}\right)\Gamma \left(\chi_{A},\delta_{A}\sqrt{\frac{\gamma_{th}}{{\bar{\rho }}_{S}}}\right)}{\Gamma \left(\chi_{A}\right)\Gamma \left(m_{h}\right)}.$$

Combining Equations (12b) and (13a), $B_{2}$ can be written as:

(A3) $$\begin{array}{cc} B_{2}= & \mathrm{Pr}\left(\rho_{I}{\left|A\right|}^{2}>\gamma_{th}{\left|h\right|}^{2},{\left|h\right|}^{2}>\frac{\rho_{I}}{{\bar{\rho }}_{S}}\right)\\ = & \int_{\frac{\rho_{I}}{{\bar{\rho }}_{S}}}^{\infty }f_{{\left|h\right|}^{2}}\left(x\right)\left[1-F_{{\left|A\right|}^{2}}\left(\frac{\gamma_{th}x}{\rho_{I}}\right)\right]dx\\ = & \frac{\mu^{m_{h}}}{\Gamma \left(m_{h}\right)}\int_{\frac{\rho_{I}}{{\bar{\rho }}_{S}}}^{\infty }e^{-\mu x}x^{m_{h}-1}\left[1-\frac{\gamma \left(\chi_{A},\delta_{A}\sqrt{\frac{\gamma_{th}x}{\rho_{I}}}\right)}{\Gamma \left(\chi_{A}\right)}\right]dx. \end{array}$$

With the aid of (Equations (8.354.1) and (3.351.2) in [46]), we have

(A4) $$\begin{array}{cc} B_{2}= & \frac{\mu^{m_{h}}}{\Gamma \left(m_{h}\right)}\left[\frac{1}{\mu^{m_{h}}}\Gamma \left(m_{h},\frac{\rho_{I}}{{\bar{\rho }}_{S}}\mu \right)-\int_{\frac{\rho_{I}}{{\bar{\rho }}_{S}}}^{\infty }e^{-\mu x}x^{m_{h}-1}\right.\\ \; & \left.\times \frac{\gamma \left(\chi_{A},\delta_{A}\sqrt{\frac{\gamma_{th}x}{\rho_{I}}}\right)}{\Gamma \left(\chi_{A}\right)}\right]dx\\ = & \frac{\mu^{m_{h}}}{\Gamma \left(m_{h}\right)}\left[\frac{1}{\mu^{m_{h}}}\Gamma \left(m_{h},\frac{\rho_{I}}{{\bar{\rho }}_{S}}\mu \right)-\sum_{q=0}^{\infty }\frac{{\left(-1\right)}^{q}\delta_{A}^{\chi_{A}+q}}{q!\Gamma \left(\chi_{A}\right)}\right.\\ \; & \left.\times \frac{\gamma_{th}^{\frac{\chi_{A}+q}{2}}}{\left(\chi_{A}+q\right)\rho_{I}^{\frac{\chi_{A}+q}{2}}}\int_{\frac{\rho_{I}}{{\bar{\rho }}_{S}}}^{\infty }e^{-\mu x}x^{\frac{\chi_{A}+q+2m_{h}-2}{2}}\right]dx\\ = & \frac{1}{\Gamma \left(m_{h}\right)}\left[\Gamma \left(m_{h},\frac{\rho_{I}}{{\bar{\rho }}_{S}}\mu \right)-\sum_{q=0}^{\infty }\frac{{\left(-1\right)}^{q}\delta_{A}^{\chi_{A}+q}}{q!\Gamma \left(\chi_{A}\right)\left(\chi_{A}+q\right)}\right.\\ \; & \frac{\gamma_{th}^{\frac{\chi_{A}+q}{2}}}{\rho_{I}^{\frac{\chi_{A}+q}{2}}\mu^{\frac{\chi_{A}+q-2}{2}}}\left.\times \Gamma \left(\frac{\chi_{A}+q+2m_{h}}{2},\frac{\rho_{I}\mu }{{\bar{\rho }}_{S}}\right)\right]. \end{array}$$

Substituting Equations (A2) and (A4) into Equation (16), the OP at SU regime can be obtained as given in Equation (17).

With that, the proof of Proposition 1 is concluded.

## Appendix B. Proof of Proposition 2

The expression for considered ER $C_{SU}$ is formulated as:

(A5) $$\begin{array}{cc} C_{SU}= & E\left\{\log_{2}\left(1+\underset{X}{\underset{︸}{\rho_{S}{\left|A\right|}^{2}}}\right)\right\}\\ = & \frac{1}{2\ln2}\int_{0}^{\infty }\frac{1-F_{X}\left(x\right)}{1+x}dx. \end{array}$$

From Equation (17), $F_{X}\left(x\right)$ can be calculated as:

(A6) $$F_{X}\left(x\right)=1-V_{1}-V_{2},$$

where $V_{1}=\frac{\gamma \left(m_{h},\frac{\mu \rho_{I}}{{\bar{\rho }}_{S}}\right)\Gamma \left(\chi_{A},\delta_{A}\sqrt{\frac{x}{{\bar{\rho }}_{S}}}\right)}{\Gamma \left(\chi_{A}\right)\Gamma \left(m_{h}\right)}$ and $V_{2}=I_{1}-\sum_{q=0}^{\infty }\frac{{\left(-1\right)}^{q}\delta_{A}^{\chi_{A}+q}x^{\frac{\chi_{A}+q}{2}}}{q!\left(\chi_{A}+q\right)\rho_{I}^{\frac{\chi_{A}+q}{2}}}I_{2}$ in which $I_{1}=\frac{1}{\Gamma \left(m_{h}\right)}\Gamma \left(m_{h},\frac{\rho_{I}}{{\bar{\rho }}_{S}}\mu \right)$ and $I_{2}=\frac{\Gamma \left(\frac{\chi_{A}+q+2m_{h}}{2},\frac{\rho_{I}\mu }{{\bar{\rho }}_{S}}\right)}{\Gamma \left(m_{h}\right)\Gamma \left(\chi_{A}\right)\mu^{\frac{\chi_{A}+q-2}{2}}}$.

Substituting Equation (A6) into Equation (A5), $C_{SU}$ is rewritten as:

(A7) $$\begin{array}{cc} C_{SU}= & \frac{1}{2\ln2}\left[\int_{0}^{\infty }\frac{\Delta_{1}\Gamma \left(\chi_{A},\delta_{A}\sqrt{\frac{x}{{\bar{\rho }}_{S}}}\right)}{1+x}dx\right.\\ \; & \left.+\int_{0}^{\infty }\frac{1}{1+x}\left(I_{1}-\sum_{q=0}^{\infty }\Delta_{2}x^{\frac{\chi_{A}+q}{2}}\right)dx\right], \end{array}$$

where $\Delta_{1}=\frac{\gamma \left(m_{h},\frac{\mu \rho_{I}}{{\bar{\rho }}_{S}}\right)}{\Gamma \left(\chi_{A}\right)\Gamma \left(m_{h}\right)}$ and $\Delta_{2}=\frac{{\left(-1\right)}^{q}\delta_{A}^{\chi_{A}+q}}{q!\left(\chi_{A}+q\right)\rho_{I}^{\frac{\chi_{A}+q}{2}}}I_{2}$.

We set $t=\frac{4}{\pi }\arctan\left(x\right)-1\Rightarrow \tan\left(\frac{\pi \left(t+1\right)}{4}\right)=x\Rightarrow \frac{\pi }{4}\sec^{2}\left(\frac{\pi }{4}\left(t+1\right)\right)dt=dx$, and we have $C_{SU}$ given by:

(A8) $$\begin{array}{cc} C_{SU}= & \frac{\pi }{8\ln2}\left[\int_{-1}^{1}\frac{\Delta_{1}\Xi \left(t\right)\Gamma \left(\chi_{A},\delta_{A}\sqrt{{\bar{\rho }}_{S}^{-1}g\left(t\right)}\right)}{1+g\left(t\right)}dt\right.\\ \; & \left.+\int_{-1}^{1}\frac{\Xi \left(t\right)}{1+g\left(t\right)}\left(I_{1}-\sum_{q=0}^{\infty }\Delta_{2}g{\left(t\right)}^{\frac{\chi_{A}+q}{2}}\right)dt\right], \end{array}$$

where $g\left(t\right)=\tan\left(\frac{\pi \left(t+1\right)}{4}\right)$, $\Xi \left(t\right)=\sec^{2}\left(\frac{\pi }{4}\left(t+1\right)\right)$ and sec2x=11cos2xcos2x.

Regrettably, obtaining a closed-form expression for $C_{SU}$ is a challenging task, but an accurate approximation can be attained. By employing the Gaussian–Chebyshev quadrature (Equation (25.4.38) in [47]), it can be achieved as follows:

(A9) $$\begin{array}{cc} C_{SU}\approx & \frac{\pi^{2}}{8U\ln2}\left[\sum_{u=1}^{U}\frac{\Delta_{1}\sqrt{1-\xi_{u}^{2}}\Xi \left(\xi_{u}\right)}{1+g\left(\xi_{u}\right)}\right.\\ \; & \times \Gamma \left(\chi_{A},\delta_{A}\sqrt{{\bar{\rho }}_{S}^{-1}g\left(\xi_{u}\right)}\right)+\sum_{u=1}^{U}\frac{\sqrt{1-\xi_{u}^{2}}}{1+g\left(\xi_{u}\right)}\\ \; & \left.\times \Xi \left(\xi_{u}\right)\left(I_{1}-\sum_{q=0}^{\infty }\Delta_{2}g{\left(\xi_{u}\right)}^{\frac{\chi_{A}+q}{2}}\right)\right]. \end{array}$$

Here, $\xi_{u}=\cos\left(\frac{2u-1}{2U}\pi \right)$. The proof of Proposition 2 is concluded.
